# Reference‐based sensitivity analysis for time‐to‐event data

**DOI:** 10.1002/pst.1954

**Published:** 2019-07-15

**Authors:** Andrew Atkinson, Michael G. Kenward, Tim Clayton, James R. Carpenter

**Affiliations:** ^1^ Department of Medical Statistics London School of Hygiene and Tropical Medicine London UK; ^2^ Department of Infectious Diseases Bern University Hospital University of Bern, Bern Switzerland; ^3^ Ashkirk Scotland UK; ^4^ MRC Clinical Trials Unit, University College London London UK

**Keywords:** missing data, MNAR, multiple imputation, sensitivity analysis, time to event

## Abstract

The analysis of time‐to‐event data typically makes the censoring at random assumption, ie, that—conditional on covariates in the model—the distribution of event times is the same, whether they are observed or unobserved (ie, right censored). When patients who remain in follow‐up stay on their assigned treatment, then analysis under this assumption broadly addresses the de jure, or “while on treatment strategy” estimand. In such cases, we may well wish to explore the robustness of our inference to more pragmatic, de facto or “treatment policy strategy,” assumptions about the behaviour of patients post‐censoring.

This is particularly the case when censoring occurs because patients change, or revert, to the usual (ie, reference) standard of care. Recent work has shown how such questions can be addressed for trials with continuous outcome data and longitudinal follow‐up, using *reference‐based multiple imputation*. For example, patients in the active arm may have their missing data imputed assuming they reverted to the control (ie, reference) intervention on withdrawal. Reference‐based imputation has two advantages: (a) it avoids the user specifying numerous parameters describing the distribution of patients' postwithdrawal data and (b) it is, to a good approximation, *information anchored*, so that the proportion of information lost due to missing data under the primary analysis is held constant across the sensitivity analyses. In this article, we build on recent work in the survival context, proposing a class of reference‐based assumptions appropriate for time‐to‐event data. We report a simulation study exploring the extent to which the multiple imputation estimator (using Rubin's variance formula) is information anchored in this setting and then illustrate the approach by reanalysing data from a randomized trial, which compared medical therapy with angioplasty for patients presenting with angina.

## 1 INTRODUCTION

Survival analysis is often used to model time‐to‐event data in observational and clinical studies. Event times are sometimes not observed and these are referred to as censored at the patient's last follow‐up. Such “intercurrent events” happen for many reasons, eg, withdrawal from treatment, loss to follow up, or because the end of funded follow‐up is reached. Censored patients cannot be ignored; they have important information to convey, and this additional information has to be included in the analysis.

By convention, it is usually assumed that such missing event times are censored at random (CAR). Defined analogously to missing at random (MAR), CAR assumes that, conditional on fully observed covariates in the model, the event time process is independent of the censoring time process. Standard maximum (partial) likelihood methods provide valid parameter estimates and associated standard errors under CAR.

While this may be appropriate for the end of funded follow‐up, in many settings, we will want to explore the robustness of our inferences to informative censoring (censoring not at random). Such sensitivity analyses should be considered when we suspect that the assumption of independence between censoring and the failure time may not hold for at least some of the patients. They should establish whether the conclusions from the study are robust to plausible departures from CAR. Both the National Research Council (NRC) and the European Medicines Agency (EMA) recognize the importance of such sensitivity analysis, for example: the NRC report from 2010 states “…sensitivity analyses should be part of the primary reporting of findings from clinical trials. Examining sensitivity to the assumptions about missing data mechanisms should be a mandatory component of reporting…,”^1^ with the EMA echoing this sentiment “…sensitivity analysis should show how different assumptions influence the results obtained….”^2^ Most recently, the proposed addendum to the ICH E9 guideline on estimands and sensitivity analysis^3^ states in §A.5.2.2 “missing data require particular attention in a sensitivity analysis because the assumptions underlying any method may be hard to justify and impossible to test.”

These views have led to a range of methodological developments. For example, Scharfstein et al^4^ initially proposed a semiparametric selection model and subsequently refined their methodology in a number of papers.^5^, ^6^, ^7^, ^8^ Siannis^9^ builds on this work, developing “local sensitivity analysis” for time‐to‐event data.^10^, ^11^ Bradshaw et al^12^ investigate nonignorably missing covariates using a full Bayesian approach, extending earlier formulations of survival analysis for CAR data (eg, Ibrahim et al^13^). Bivariate and frailty models for explicitly linking the censoring and failure mechanisms are investigated in the papers by Emoto and Matthews,^14^ Thiébaut et al,^15^ and Huang and Wolfe.^16^ Methods relaxing the CAR assumption using the Kaplan‐Meier product limit have also been developed (eg, Kaciroti et al^17^).

Although these methods are elegant, they have not been extensively used in trials. However, under censoring at random, we can use multiple imputation to impute the unobserved event times in a principled manner, yielding statistically valid inferences that are equivalent to those from the corresponding (partial) likelihood. The advantage of using multiple imputation is that we can readily modify our imputation model to explore the sensitivity of inferences to departures from CAR. This has the potential to provide a flexible approach for targeting clinically relevant estimands, such as those discussed by Mallinckrodt et al.^18^

In this article, we describe how sensitivity analysis using reference‐based imputation, proposed by Carpenter et al^19^ in the longitudinal continuous data setting, can be extended to time‐to‐event data. In the continuous data setting, Carpenter et al proposed that, once the estimand is defined, patients should be followed up until they deviate from the protocol in a way that is relevant to the estimand. Subsequently, we assume for present purposes their data are missing. The primary analysis then needs to make an assumption about the distribution of each patient's missing values given their observed values. A reasonable assumption for the primary analysis may be “MAR,” that is, that the conditional distribution of postdeviation given predeviation data can be estimated from patients with similar predeviation profiles (eg, patients from the same treatment arm) who did not deviate. Then, to perform the sensitivity analyses, instead of the analyst specifying a (potentially large number) of sensitivity parameters, missing values are imputed “by reference” to other groups of patients. For example, patients in the active arm may be imputed “by reference” to those in the control arm.

Such methods display the natural advantages of pattern mixture models. For example, if multiple nonrandom interventions (NRIs) occur—as in adjuvant cancer trials—then in principle, we can handle this by changing the subsequent hazard to that estimated from the relevant reference group (assuming we have data from suitable patients). Then, once again, we can use a multiple imputation approach for inference.

This broad approach has proved attractive, as reflected by the recent literature. For example, Tang^20^ proposes an extension of control‐based imputation to longitudinal binary and ordinal data, working on the scale of the linear predictor, and give an MCMC algorithm implementing the approach. Keene et al^21^ show how to use controlled imputation for sensitivity analysis under a negative binomial for recurrent events; in a similar setting, Gao et al^22^ show how to use controlled imputation with a piecewise exponential model. In the survival setting, Lu et al^23^ compared two approaches to sensitivity analysis with controlled multiple imputation, while Lipkovich et al^24^ propose an approach to tipping point analysis with survival data. Zhao et al use nonparametric multiple imputation to investigate potentially informative censoring, including a reference‐based approach (section 6.3 of Zhao et al^25^).

In this paper, we show first how each of the proposals in Carpenter et al^19^ may be applied in the context of time‐to‐event data. This includes the proposals of Lu et al^23^ and Lipkovich et al.^24^ We show how imputation and inference can be performed using Rubin's rules. Then, in contrast to a number of recent papers, we demonstrate by simulation that Rubin's rules give inferences that are approximately *information anchored* relative to the primary analysis. This property, which can be theoretically demonstrated in certain special settings^26^, ^27^ means that regulators and industry can be confident that, relative to the primary analysis, the sensitivity analyses are neither unobtrusively injecting or removing statistical information. We believe that keeping a “level playing field” in this way is important in regulatory work. For illustration, we consider a clinical trial in cardiovascular disease. In these data, we initially censor follow‐up at the first nonrandomized intercurrent event. We then impute the event times under a specific, realistic, de facto (intention to treat or treatment policy) assumption. We then find that our imputed results are consistent with the actual de facto observed event time data, so providing empirical justification for the approach.

The article proceeds as follows. Section 2 introduces the cardiovascular trial RITA‐2, which we use to illustrate the approach. Our proposals for reference‐based imputation are set out in Section 3. We review the concept of information anchoring in Section 4 and present the results of a simulation study. The example is revisited in Section 5, and we close with a discussion in Section 6.

## 2 THE RITA‐2 STUDY

The second randomized Intervention Treatment of Angina^28^, ^29^ randomized 1018 eligible coronary artery disease patients from the United Kingdom and Ireland to receive either Percutaneous Transluminal Coronary Angioplasty (PTCA, n = 504) or continued medical treatment (n = 514). Those patients randomized to angioplasty received the intervention in the first 3 months. The primary endpoint of the study was a composite of all cause death and definite nonfatal myocardial infarction.

This was a pragmatic trial, so in the course of the follow‐up patients received further procedures according to clinical need. These were either PTCA or when necessary a coronary artery bypass graft (CABG). In the PTCA arm, 17.0% of patients had a second PTCA, while 12.7% had a CABG. By contrast, on the medical arm 27% had a nonrandomized PTCA (this was typically the first nonrandomized intervention) and 12.3% had a CABG.

**Figure 1:**
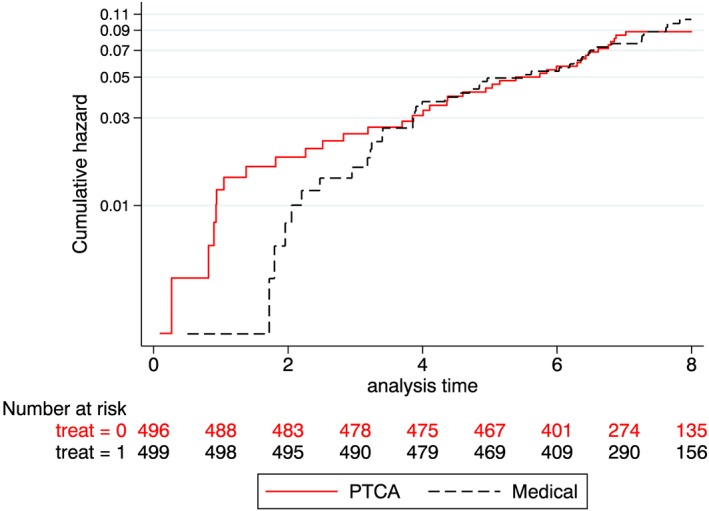
RITA‐2 trial–Nelson‐Aalen cumulative hazard survival plots for all cause mortality (up to 8 y only 18 patients lost to follow‐up)

Figure 1 shows the log‐cumulative hazard for all cause mortality, with patients censored at the end of study follow‐up. This illustrates the study's main conclusion, that an initial policy of PTCA was associated with greater improvement in angina symptoms, and that the increased risk of performing PTCA should be offset against these benefits. This is consistent with the top row of Table 2, which presents the results from fitting a proportional hazards model to the data from the original study with 8 years of follow‐up. As this is an average ratio between the hazards for the medical and PTCA arms over this period, it is close to 1.

Here, we take all cause death as the event and compare two approaches with estimating the de facto (ie, treatment policy) effect.

The first approach simply analyses the observed data, which we can do directly because follow up was continued after NRIs until the end of the study.

The second analysis targets the same question using our proposed reference‐based sensitivity approach. Specifically, we artificially censor follow‐up in the medical arm at the time of the first NRI. Since these NRIs are predominately PTCAs, it is plausible that we will get similar results to our first analysis if we impute the missing event times as if, from that point onwards, patients experienced the hazard of the PTCA arm—in other words they “jumped to PTCA”.

If this second, “jump to PTCA”, approach gives a similar answer to the first, then we have empirically illustrated its validity in our setting. This in turn builds confidence that—in similar settings where post‐NRI event times are missing—our approach provides a plausible, practical way forward. This supports its use in such settings.

First, in the next section, we develop our approach.

## 3 REFERENCE‐BASED SENSITIVITY ANALYSIS FOR SURVIVAL DATA

Consider a two arm trial, with patients randomly assigned to either an active treatment, or a reference treatment (eg, placebo or standard of care), with a time‐to‐event outcome. Typically in such studies, a number of patients in each arm will be censored at scheduled end of follow‐up, and this is plausibly censoring at random. For simplicity, here we do not consider this cause of censoring. Instead, we suppose that a number of patients in the active arm are censored not at random (in trials, examples of this would be nonrandomized interventions, or other intercurrent events). Following Carpenter et al,^19^ we describe a number of options for imputing the missing event times.

Let *i*=1,…,*n* index patients and *t*_*i*_ the event time. *t*_*i*_ is only observed if *t*_*i*_<*c*_*i*_, where *c*_*i*_ is the censoring time. Define

$$\left\{\begin{array}{ll} x_{i}=1 & \text{if patient}\;i\;\text{is in the active group, and}\\ x_{i}=0 & \text{if patient}\;i\;\text{is in the reference group} \end{array}\right.,$$

and, for times *t*<*c*_*i*_ let the hazard at time *t* for patient *i* be $h(t;x_{i},\beta )=h_{0}(t)\exp(\beta x_{i}),$ where *h*_0_(*t*) is the hazard in the reference group. We assume proportional hazards so that *β* is the log hazard ratio of treatment.

For patient *i*, censored at *c*_*i*_, we now define their hazard as follows:

(1) $$h_{i}(t)=\left\{\begin{array}{ll} h_{0}(t)\exp(\beta x_{i}) & t\leq c_{i}\\ h_{post,i}(t) & t>c_{i} \end{array}\right.,$$

where the index *post* denotes the postcensorship hazard.

Once we specify a form for *h*_*post*,*i*_ we can apply multiple imputation to event times for all censored patients, then fit our substantive model to each imputed data set before combining the results for final inference using Rubin's rules.

In the next subsection, we describe how to impute the missing event times under censoring at random, that is when we assume $h_{post,i}(t)=h_{0}(t)\exp(\beta x_{i}).$ In this case, our inferences should be equivalent (up to Monte Carlo error) to those from maximum (partial) likelihood. We then go on to consider alternative specifications for the postcensoring hazard.

### 3.1 Imputation under CAR

Our approach follows that in chapter 8.1.3 of Carpenter and Kenward.^30^ First, we need to choose our substantive model. For our development, this will be a proportional hazards model. Imputing the missing events under the Cox proportional hazards model involves drawing proper imputations from the baseline hazard, *h*_0_(*t*). This is possible (see Jackson et al^31^) but entails additional computational complications. Instead, for our development, we take the Weibull proportional hazards model as substantive and imputation model. This is sufficiently flexible for many applications; in other settings, we suggest using a flexible spline as a parametric model for the baseline hazard, again with proportional hazards (eg, Royston and Lambert^32^; Royston and Parmar^33^).

Imputation proceeds as follows:

1 Under censoring at random, fit the Weibull model to the observed data, obtaining the maximum likelihood estimates of the parameters $\hat{\beta }$ and its covariance matrix, $\hat{\sum }.$
  For *k*=1,…,*K* imputations
  - Draw $\tilde{\beta }∼N(\hat{\beta },\hat{\sum }).$
  - For each patient with censored data, draw their event time from $h_{i}(t;\tilde{\beta }),$ by equating the conditional survivor function, $S(t_{i}|t_{i}>c_{i},x_{i},\tilde{\beta })$ to a uniform distribution and solving for *t*_*i*_.
    Under our Weibull model, we draw *u*_*i*_∼*U*[0,1] and solve
    $$S(t_{i}|t_{i}>c_{i},x_{i},\tilde{\beta })=\frac{S(t_{i};x_{i},\tilde{\beta })}{S(c_{i};x_{i},\tilde{\beta })}=u_{i},$$
    which has a simple closed form solution.
2. Fit the substantive model to each imputed data sets resulting in *K* estimates of the log hazard ratio and combine these using Rubin's rules.

### 3.2 Proposals for reference‐based imputation under censoring not at random (CNAR)

We now give some suggestions for reference‐based imputation under CNAR. To keep the presentation simple, we focus on imputing censored outcomes in the intervention group (*x*_*i*_=1); although the approach is quite general. Without loss of generality we assume those censored on the reference arm are CAR throughout. For each method, we define a different reference group for the postcensorship hazard and briefly discuss its plausibility in practice.

#### 3.2.1 Jump to Reference

**Figure 2:**
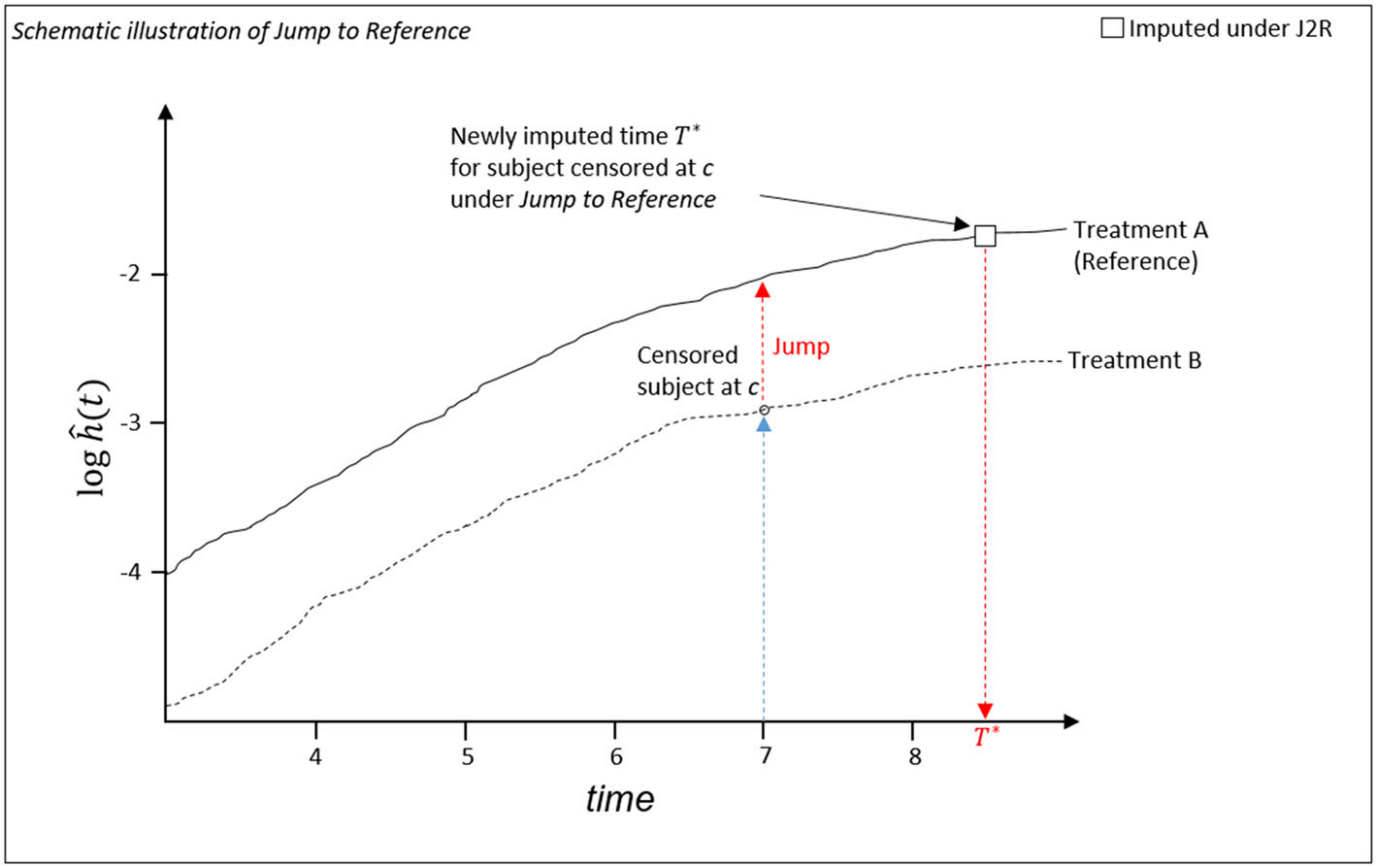
Time‐to‐event data–Jump to Reference

Under Jump to Reference, an active arm patient censored at *c* switches to the reference arm hazard for *t*>*c*. This is schematically illustrated in Figure 2, where a patient is censored at *c*, and then the J2R method imputes a new event time at time *T* ^∗^. Note that the reference hazard is estimated from the reference arm assuming censoring at random.

When the active treatment has a lower hazard, Jump to Reference models a scenario in which a patient discontinuing (deviating) from the active treatment experiences no further benefit but instead reverts to the hazard in the control (reference) group. For example, this might occur when treatment B is a higher dose of treatment A, a patient randomized to treatment B has to discontinue the treatment due to increased toxicity, so their dose and hazard then drop to that of the reference treatment A.

As usual, once a patient's postcensoring hazard is specified, the event time is imputed by generating a new time *T* ^∗^.

Note that, because we are now considering survival data, this method is equivalent to the “copy reference” approach in the longitudinal data setting. Also, under this method, we can choose for the patient to jump to the hazard in the reference group at any time *t* during the follow‐up, but *t*=*c* is most natural.

#### 3.2.2 Last hazard carried forward

Under this assumption, when a patient in the active arm is censored at *c*, their postcensorship hazard remains what it was at that *c*, ie,  *h*_*post*,*i*_(*t*)=*h*_*i*_(*c*_*i*_).

#### 3.2.3 Copy increments in reference hazard (CIR)

Here, the postcensoring hazard copies the increments in the reference hazard, so that

$$h_{post,i}(t|t>c_{i})=\frac{h_{act}(c_{i})}{h_{ref}(c_{i})}h_{ref}(t),$$

where *h*_*act*_(*t*) is the hazard on the active arm at time *t*.

Under proportional hazards (1), this is equivalent to censoring at random; under nonproportional hazards, it will differ.

#### 3.2.4 Delta method

Here, a patient's postcensoring hazard is a multiple of the hazard in their treatment group, ie, 

$$h_{post}(t|t>c_{i})=\Delta h_{act}(t).$$

##### 3.2.4.1 Comments

As in the case of longitudinal data, the delta‐method is the only approach that requires the user to specify a sensitivity parameter. This has the potential advantage that a “tipping point” analysis can be performed, whereby Δ is moved away from 1 until the conclusions change. Alternatively, we may seek expert opinion on Δ, but this may be controversial.^34^, ^35^, ^36^

Many analyses of trials assume that hazards are proportional. The proportional hazards assumption can be checked visually by plotting the Schoenfeld residuals.^37^ This may be complemented with the Grambsch‐Therneau test^38^—although the test is often not definitive and can be insensitive to certain forms of nonproportionality. The recent publication by Keogh and Morris reviews and discusses methods for determining if the proportional hazards assumption holds,^39^ and Ng'andu's presents empirical comparisons of methods for assessing proportional hazards.^40^

Although the hazard ratio can always be interpreted as the average hazard ratio over the follow‐up, nevertheless, this single summary is increasingly being challenged in the oncology setting,^41^, ^42^ and the restricted mean survival time has been proposed as an alternative summary measure. Our proposed approach can be directly applied when the effect measure is RMST. Nevertheless, RMST is not a panacea because it inevitably involves a somewhat arbitrary choice of time horizon. Piece‐wise proportional hazards models may also be considered, along with nonparametric methods which do not strictly require proportional hazards.^25^

Assuming that the primary analysis is proportional hazards, apart from CAR, the methods above imply a mixture of hazards in the active arm, which therefore strictly violates the PH assumption. In applications, we have not found this to be a practical issue, not least because the HR remains a valid estimate of the average hazard for the period studied. However, if there is concern that this may be inappropriate, for example, because a test of departure from PH is significant, alternative numerical and graphical summary measures may be preferred. By definition, under a non‐PH model primary analysis model, this is not an issue.

## 4 SIMULATION STUDY

There has been some discussion of the use of Rubin's rules to estimate the variance for reference‐based multiple imputation—with the alternative being the empirical standard error from fitting the model to bootstrapped data (or theoretical approximations to this^43^). In the context of longitudinal data, Carpenter et al^44^ sketch that, because distributional information is borrowed under reference‐based methods, the standard likelihood calculation results in an artificial gain in statistical information about the treatment effect, relative to what we would expect to see if the missing data were able to be actually observed, and their distribution corresponded to that under the reference‐based assumption. By contrast, they propose, and Cro et al^43^ prove, that for continuous longitudinal data using Rubin's rules is—to a good approximation—*information anchored*. This means that reference‐based imputation using Rubin's rules in the conventional way approximately preserves the fraction of information lost due to missing data across each of the assumptions. In practice, an information anchored analysis means that standard errors and widths of confidence intervals for analyses remain approximately constant. If one of the assumptions is chosen for the primary analysis (typically MAR), this means that the information about the treatment effect lost due to missing data is constant across the primary and sensitivity analyses.

For log‐normal time‐to‐event data, we have shown that reference‐based sensitivity analyses using multiple imputation are information anchored.^45^ More general theoretical results are challenging. In this section, we therefore explore by simulation the extent to which reference‐based imputation for time‐to‐event data are information anchored. We do this by simulating time‐to‐event data from a two arm trial, with active and reference (ie, control) arms. Without loss of generality, we only censored patients in the active arm; all event times in the reference arm are observed.

We simulated event times from an exponential distribution, with control arm hazard *h*(*t*)=0.01, and hazard ratio *β*, using the approach described by Bender et al.^46^ Data in the active arm were CAR and then imputed assuming (a) censoring at random and (b) Jump to Reference. We varied the active arm censoring levels from 0% to 80% and explored three different sample sizes: *n*=125, 250 and 500 in each arm. For all the results presented below, we used *K*=50 imputations and 1000 replications.

To each simulated data set, we fitted the Weibull proportional hazards model:

(2) $${ĥ}_{i}(t)=\kappa t^{\kappa -1}\exp(\alpha +\hat{\beta }x_{i}).$$

We focus on the treatment estimate $\hat{\beta }.$

For the first scenario, the hazard ratio used to generate the data is *β*=0.8 (log hazard ratio −0.22314) with 250 patients in each arm, giving a power of 0.7 when there is no censoring.

Table 1 shows the results. The second row of Table 1 shows the results when there is no censoring. The mean of the estimates of *β* across the *S*=1000 replications,

(3) $$\hat{E}[\hat{\beta }]=\frac{1}{S}\sum_{s=1}^{1000}{\hat{\beta }}_{s},$$

is −0.22695. Over the *S* replications, the mean value of the asymptotic variance estimate, calculated as the inverse of the observed information,

(4) $$\hat{E}[{\hat{V}}_{inf}(\hat{\beta })]=\frac{1}{S}\sum_{s=1}^{1000}{\hat{V}}_{inf}({\hat{\beta }}_{s}),$$

is 0.00797, while, letting ${\hat{\beta }}_{.}=\sum_{s=1}^{S}{\hat{\beta }}_{s},/S$ be the usual empirical variance estimate,

(5) $${\hat{V}}_{emp}(\hat{\beta })=\frac{1}{\left(S-1\right)}\sum_{i=1}^{1000}{\left({\hat{\beta }}_{s}-{\hat{\beta }}_{.}\right)}^{2},$$

is 0.00807. Therefore, we see that when there is no censoring, the mean of ${\hat{\beta }}_{s}$ over the *S*=1000 replications is unbiased, and the theoretical and empirical variance estimates agree as expected.

**Table 1:** Simulation results: exponential data generating process, 250 patients in each arm, censoring in the active arm only; Weibull analysis and imputation model, *S*=1000 replications

Column: 1	2	3	4	5	6	7	8
Censoring %	True *β*	$\hat{E}[\hat{\beta }]$ (Censored Data Recreated	$\hat{E}[{\hat{\beta }}_{MI}]$	$\hat{E}[{\hat{V}}_{inf}(\hat{\beta })]$ (Censored Data Recreated	${\hat{V}}_{emp}(\hat{\beta })$ (Censored Data Recreated	$\hat{E}[{\hat{V}}_{RR}({\hat{\beta }}_{MI})]$	${\hat{V}}_{emp}({\hat{\beta }}_{MI})$
(Active Arm)		Under Current Assumption)		Under Current Assumption)	Under Current Assumption)		
No censoring	−0.22314	−0.22695		0.00797	0.00807		
Analysis assuming
Censoring at Random
10%	−0.22314	−0.22679	−0.22821	0.00797	0.00813	0.00850	0.00844
20%	−0.22314	−0.22692	−0.22933	0.00797	0.00801	0.00918	0.00912
30%	−0.22314	−0.22690	−0.23009	0.00796	0.00820	0.01006	0.00985
40%	−0.22314	−0.22620	−0.23086	0.00797	0.00784	0.01114	0.01093
50%	−0.22314	−0.22726	−0.23146	0.00797	0.00838	0.01244	0.01227
60%	−0.22314	−0.22497	−0.22866	0.00798	0.00798	0.01460	0.01456
80%	−0.22314	−0.22627	−0.23433	0.00798	0.00808	0.02507	0.02483
Analysis assuming
Jump‐to‐Reference
10%	−0.42608	−0.20751	−0.20833	0.00793	0.00784	0.00830	0.00703
20%	−0.18232	−0.18727	−0.18941	0.00792	0.00793	0.00882	0.00621
30%	−0.16127	−0.16615	−0.16807	0.00790	0.00796	0.00952	0.00536
40%	−0.13976	−0.14452	−0.14639	0.00790	0.00801	0.01046	0.00468
50%	−0.11778	−0.12274	−0.12559	0.00790	0.00819	0.01147	0.00424
60%	−0.09531	−0.09508	−0.09972	0.00793	0.00827	0.01298	0.00382
80%	−0.04879	−0.04956	−0.05521	0.00803	0.00817	0.01610	0.00350

**Table 2:** RITA‐2 analysis: estimated all cause mortality hazard ratios comparing PTCA with the medical intervention based on the original study data (top) and the emulated “Jump to PTCA” de‐facto scenario (bottom); hazard ratio > 1 indicating the risk is higher on the medical arm

Estimand	Hazard Ratio (95% CI)	*P* Value
De‐facto analysis of study data	1.02 (0.67‐1.57)	.93
Emulated de‐facto analysis:		
Medical arm patients are censored at their first
nonrandomized intervention and their event times	1.15 (0.75‐1.55)	.49
are imputed under “Jump to PTCA arm”.		

We now explore what happens when data are CAR in the active arm only. When this happens, we need to make an (untestable) assumption about the censored data. Here, we estimate the hazard ratio by multiple imputation under this assumption.

The top half of Table 1 shows the results when we assume data are CAR and impute accordingly. We define three quantities from the multiple imputation estimates analogous to (3) to (5) above. These are, first the mean of the estimates across the *S* replications,

(6) $$\hat{E}[{\hat{\beta }}_{MI}]=\frac{1}{1000}\sum_{s=1}^{1000}{\hat{\beta }}_{s,MI},$$

second the mean of the “Rubin's rules” variance of these estimates,

(7) $$\hat{E}[{\hat{V}}_{RR}({\hat{\beta }}_{MI})]=\frac{1}{1000}\sum_{s=1}^{1000}{\hat{V}}_{RR}({\hat{\beta }}_{s,MI}),$$

and third the empirical variance of the *S* multiple imputation estimates,

(8) $${\hat{V}}_{emp}({\hat{\beta }}_{MI})=\frac{1}{\left(S-1\right)}\sum_{i=1}^{1000}{\left({\hat{\beta }}_{s,MI}-{\hat{\beta }}_{.,MI}\right)}^{2},$$

where ${\hat{\beta }}_{.,MI}=\sum_{s=1}^{1000}{\hat{\beta }}_{s,MI}/S.$

To assess the information anchoring properties, in columns 3 and 5 of Table 1, the censored data are recreated (put back) under the current assumption before the quantities are calculated. In the top half of the table, we assume censoring at random. If they are recreated under this assumption, then we get a full data set from the exponential data generating model. Therefore, in the top half of Table 1, the values in columns 3 and 4 only differ from each other by Monte Carlo variation as the proportion of censoring increases. Likewise, columns 5 and 6 only differ by Monte Carlo variation.

In column 7, we see—again as expected—that Rubin's rules variance of the imputation estimate increases as the proportion of censoring increases, and this agrees well with the empirical variance of the MI estimator.

Now consider the bottom half of Table 1. Here, when the data are censored, we assume “Jump to Reference”. As above, in columns 3 and 5, we recreate (put back) the data under this assumption. Column 3 shows that the mean treatment effect attenuates as the proportion of censoring increases, and comparing with column 2, we see there is no systematic bias. Columns 5 and 6 show that when censored data is recreated under the current assumption, the information‐based and empirical variance estimates are similar, as expected, and do not vary markedly as the proportion of censoring increases.

Now consider column 8. This shows the empirical variance of the MI estimates. Because imputation under Jump to Reference borrows information from the reference arm, the empirical variance *declines* as the proportion of censoring increases. Further, it is *less than* the variance we would see if the assumption held true and we saw the data (column 6). We therefore argue that the empirical variance in column 8 (and theoretical approximations to it) is not appropriate: using it would imply that by censoring 80% of the active arm, we *double* the statistical information about the treatment effect.

Instead, we advocate using Rubin's rules variance (column 7). We see that this increases as the proportion of censored data increases, reflecting the loss of information about the treatment effect.

**Figure 3:**
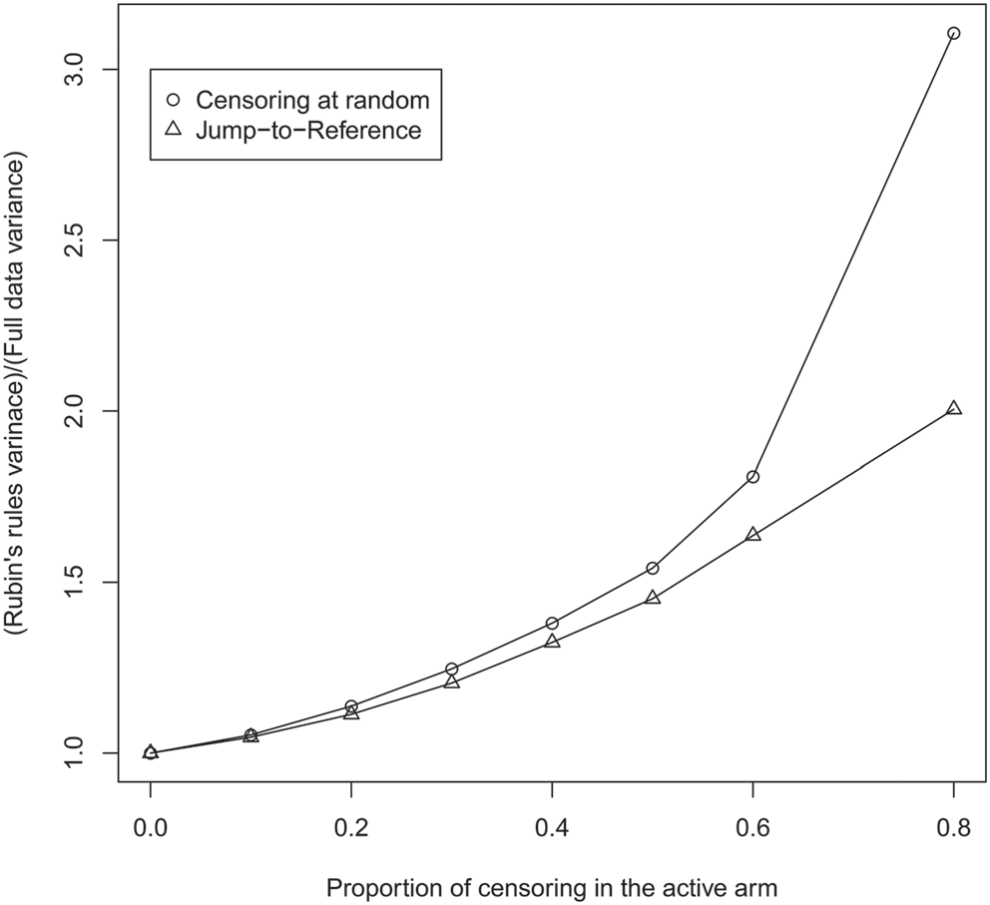
Proportionate increase in variance as censoring increases under (a) censoring at random and (b) Jump to Reference

To explore this further, as Figure 3 shows, the proportionate increase in variance (column 7 divided by column 5) under censoring at random using Rubin's rules approximates that under Jump to Reference, and this approximation is particularly good for lower proportions of censoring. As discussed above, this is what we call *information anchoring*. In other words, the proportion of information lost due to missing data is the same under the primary analysis assumption (CAR) and the sensitivity analysis assumption (J2R), at least up to a censoring level of 60% on the active arm.

**Figure 4:**
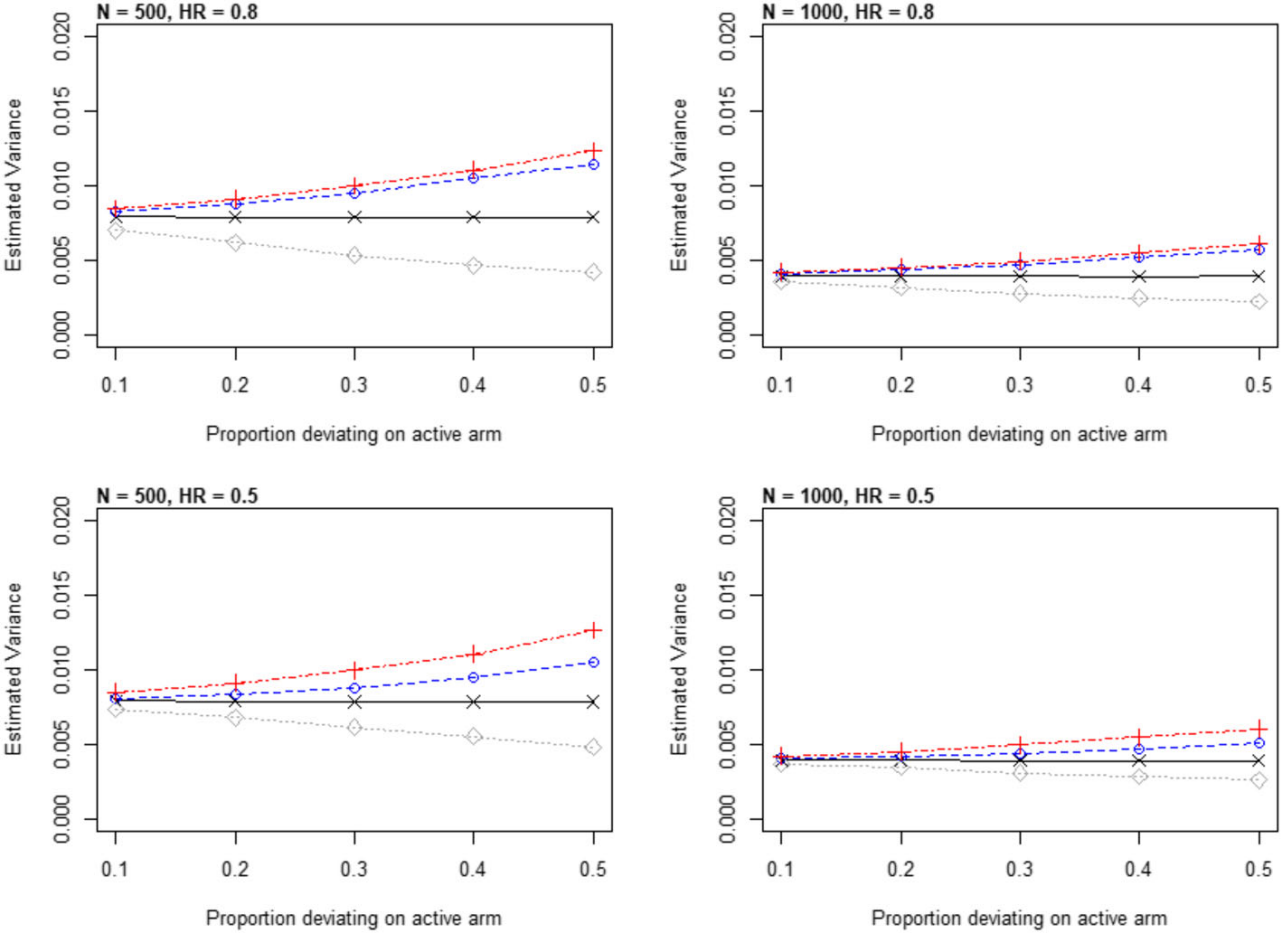
Simulation results: exploration of information anchoring for two sample sizes and two hazard ratios. For each scenario, as the proportion of active arm censoring increases, each panel shows the evolution of the variance of the estimated hazard ratio calculated in four ways: (a) −+− information anchored variance; (b) −∘− Rubin's MI variance under Jump to Reference; (c) −×− $\hat{E}[{\hat{V}}_{inf}(\hat{\beta })]$ when censored data recreated under Jump to Reference; and (d) −⋄− ${\hat{V}}_{emp}({\hat{\beta }}_{MI})$ under Jump to Reference

These results are in line with the theory for continuous data (Cro et al^43^), which shows that the approximation of Rubin's rules to information anchoring improves as the treatment effect decreases. To explore this further, we now consider additional scenarios. Figure 4 shows results for a hazard ratio of 0.5 and 0.8, for sample sizes of 250 and 500 patients in each arm.

In each panel, the horizontal lines −×− is the variance of the log‐hazard ratio when the censored data are recreated under Jump to Reference. In other words, they are derived in the same way as column 5 in Table 1. The −⋄− lines show the empirical variance of the multiple imputation estimator under Jump to Reference and are derived in the same way as column 8 in Table 1. The −∘− line denotes the Rubin's rules variance of the multiple imputation estimator under Jump to Reference (cf column 7 in Table 1), with −+− showing the information anchored variance.

Consistent with Table 1, column 8, we see that under Jump to Reference the empirical variance of the MI estimator drops below that we would obtain if we actually observed data under this assumption. However, Rubin's rules variance under CAR and Jump to Reference are very similar, especially for the smaller hazard ratio of 0.8 (top panels of Figure 4), and for smaller proportions of censoring—both more likely in trials. Thus, for reference‐based imputation of the type described here, Rubin's rules are approximately information anchored; that is, the loss of information due to missing data is approximately constant across the primary assumption about censoring and the sensitivity assumptions.

Here, we focus on the “Jump to Reference” approach since under the proportional hazards assumption the simulation results under “hazard carried forward” and “copy increments in reference” were, as might be expected, very similar to when multiply imputing under CAR (results not shown).

## 5 APPLICATION TO THE RITA‐2 DATA SET

We now return to the analysis of the RITA‐2 study. This was a pragmatic study, in which a high proportion of patients from both arms went on to have NRIs. In the medical arm, these NRIs were typically first a PTCA, with a number of patients having a second PTCA and/or a CABG. In the PTCA arm, they were typically a second PTCA and/or a CABG.

The analysis of all‐cause mortality, assuming censoring at the end of the study is censoring at random, addresses a de‐facto or “treatment policy” type of estimand. The de‐facto cumulative hazards for each arm are shown in Figure 1, and the treatment effect from an unadjusted Weibull proportional hazards model is shown in the top part of Table 2.

We now illustrate how we can emulate this analysis using reference‐based imputation. To do this, we leave the PTCA arm data unchanged. For the medical arm data, we artificially censor patients at their first NRI, and then they “Jump to Reference,” which in this context means “Jump to PTCA arm.” We implement this using the multiple imputation approach described earlier.

Specifically, the primary analysis model remains an unadjusted Weibull model. For multiple imputation under “Jump to PTCA arm”, we again use a Weibull model. In line with the recommendations from, for example, page 79 of Carpenter and Kenward,^30^ we include baseline variables predictive of the event time and associated with the censoring process. We therefore include the following covariates: treatment, sex, age, BMI, systolic blood pressure and angina grade, unstable angina, breathlessness grade, presence of a previous MI, activity level, treatment for hypertension, diabetes, smoking status, beta blockers, long acting nitrates, calcium antagonists, lipid‐lowering drugs, aspirin, ace inhibitors, and number of diseased vessels. Multiply imputed event times exceeding the maximum study period of 8 years were censored, in line with the assumptions used for the analysis in the original study.

**Figure 5:**
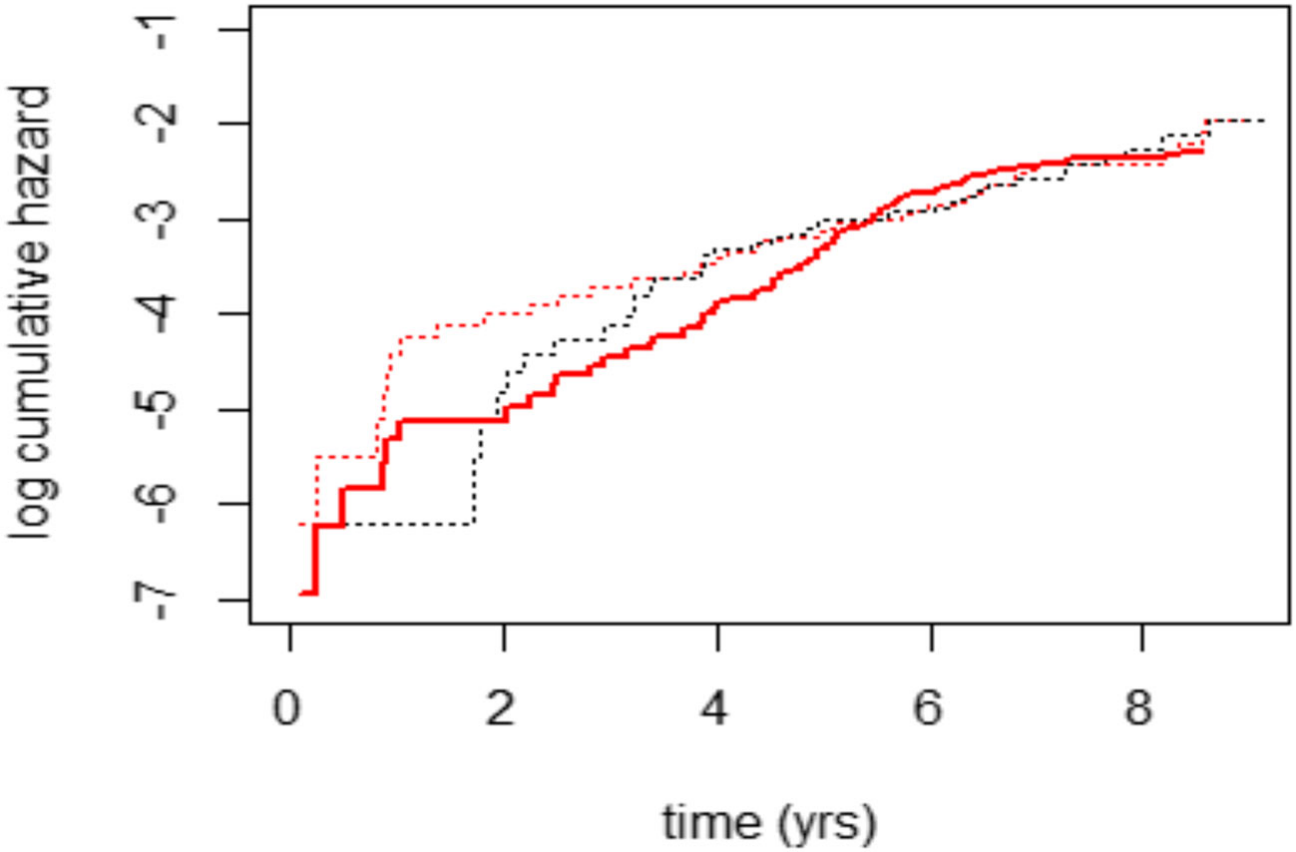
Plot of the log cumulative hazard against time with Nelson‐Aalen estimates for the PTCA arm (upper dashed, red) and medical arm (lower dashed, black). The solid (red) line shows the estimated Weibull model log cumulative hazard for the medical arm when patients are censored at their first nonrandomized intervention and “Jump to PTCA arm”

The results of emulating the de‐facto analysis by censoring medical arm patients at NRI and imputing under “Jump to PTCA arm” are shown in Table 2. We see that the emulated de‐facto results agree well with the actual de‐facto analysis, with both *P* values far from statistical significance. The solid red line in Figure 5 shows the estimated log cumulative hazard for the medical arm from fitting the Weibull model to the imputed data under “Jump to PTCA arm”. As we would hope, it is initially close to the medical arm, but as more patients on the medical arm have early NRIs, it tracks back to the PTCA arm. However, the model's proportional hazards assumption means that, in accommodating the early higher hazard in the medical arm, it under‐shoots the PCTA arm between years 2 and 5. This is why the emulated de‐facto hazard ratio is larger than the actual one in Table 2. Finally, we note that our simulations suggest our inference using Rubin's rules is information anchored—that is the fraction of information lost due to censoring is held constant across the actual and emulated analysis.

## 6 DISCUSSION

In this paper, we have further extended and evaluated the methodology of reference‐based multiple imputation from the original setting of longitudinal continuous data to time‐to‐event data. This class of methods has found increasing application in settings where a non‐trivial proportion of patients deviate from the protocol, so the analysis cannot proceed without making additional assumptions, which are not fully verifiable from the trial data.

In such settings, it is now widely recognized that we need to clearly set out assumptions for the primary analysis, and then explore the sensitivity of our inferences to analyses under alternative assumptions. Both primary and sensitivity assumptions need to be relevant and accessible; this was the motivation for the original work of Carpenter et al,^19^ where assumptions about postdeviation behaviour of patients were made by reference to other groups. A further attraction of this approach is that the primary analysis model is retained in the sensitivity analysis—being fitted to the imputed data under the sensitivity scenarios.

This approach has the advantage that it avoids what is often a key difficulty in practice—identifying values for the sensitivity parameters. This difficulty has been widely acknowledged. For example, Daniels and Hogan^47^ quote from Scharfstein et al^48^ who comment: “…the biggest challenge in conducting sensitivity analyses is the choice of one or more sensitivity parameterized functions whose interpretation can be communicated to patient matter experts with sufficient clarity….” It is therefore encouraging that reference‐based sensitivity analysis via multiple imputation has increasingly been used (see, for example, Philipsen et al,^49^ Jans et al,^50^ Billings et al,^51^ and Atri et al^52^) which motivated us to set out to systematically extend it to the time‐to‐event setting. In Section 3, we present a number of possibilities, many derived from the setting with continuous outcomes (Carpenter et al^19^). Clearly, their applicability will depend on the trial context. Our example led us to focus on “Jump to Reference,” and we anticipate this is likely to be relevant in a range of settings.

An important, but often neglected, aspect of sensitivity analysis is that the analyst has control not only of the mean but also the variability of the unobserved data. Relative to the primary analysis, it is therefore quite possible for a sensitivity analysis to increase, hold anchored, or decrease the statistical information about the treatment effect. We believe that the default choice should be to hold the statistical information constant across primary and sensitivity analyses, and that it should certainly not be increased in the sensitivity analysis. With longitudinal data, using multiple imputation with Rubin's rules achieves this (Cro et al^43^), with the best approximation when randomization is 1:1. In other settings, the theory suggests how to modify the procedure to retain a good information anchoring approximation. With time‐to‐event data, a corresponding formal proof is challenging, apart from in special circumstances.^45^ Nevertheless, the results of our simulation study closely mirror those obtained in the longitudinal setting, suggesting that similar results hold with time‐to‐event data. In particular, using the primary analysis variance estimator in the sensitivity scenarios results in the sensitivity analysis having more statistical information than the primary analysis, and this information increases as the proportion of censoring increases. This is undesirable in practice and can be avoided by using Rubin's MI rules for the sensitivity analysis.

A suitable application of the methods described here might be in the context of a superiority trial in which censoring occurs both in the active intervention and control (ie, reference) arms. The reference‐based sensitivity analysis approach could then be applied with, for example, the “Jump to Reference” method used to multiply impute events on the active arm. Rather unusually in our example, the long term follow‐up in the RITA‐2 trial allows us to compare the results of a de‐facto analysis using the observed event times with an emulated de‐facto analysis, where in the medical arm we artificially censor people at their first nonrandomized intervention and allow them to “Jump to PTCA arm”. The results are similar, providing empirical support for this approach in situations where, for whatever reason, data are censored but we wish to explore the robustness of our conclusions to the censoring at random example. Another assumption, suggested by one reviewer, would be to multiply impute data by jumping to the reference hazard at time 0. This might more adequately model the surgical risk of PTCA, followed by post‐operative improvement.

It can be argued that our illustrative example, while providing evidence of the applicability of the method, might be deemed atypical, particularly for pharmacological trials. However, other authors have presented examples of similar approaches in pharmacological settings (eg, the open label, double blinded study in Lu et al^23^). Other settings where we are exploring this approach include a sensitivity analysis in a “trial emulation” analysis of observational data using the approach proposed by Hernan and colleagues (see, eg, Hernan and Robins 53).

In many trials, patients who are lost to follow‐up are censored at their last known observation time in the analysis. As discussed on page 260 of O'Kelly and Ratitch,^54^ common practice is to make a censoring at random assumption for those on the control arm, and to investigate plausible departures from this assumption for the intervention arm. Such a sensitivity analysis contrasts the results with those from a blanket assumption of CAR at protocol violation. In other settings, if the control group is not receiving the usual standard of care treatment, it may not be the appropriate reference group; in such cases an alternative reference group within the trial may be appropriate. In all cases, it is important that the assumptions take careful account of the reason for censoring (eg, intercurrent events, end of follow‐up and sometimes death).

Notwithstanding, the flexibility of the approach, like any based on multiple imputation, caution is recommended with higher levels of censoring on an arm. Similarly, when one treatment arm has very few events then accurate estimation of the hazard is made more difficult due to the lack of information.

A limitation of our approach as set out here is that we assume proportional hazards, both for the primary analysis model and for imputing censored event times. While this is reasonable in many examples, it is not always appropriate. However, this is not an inherent limitation of the method. For example, both the primary analysis model and the reference‐based imputation model may be Royston‐Parmar models, which use a flexible spline to model the log‐cumulative hazard and therefore allow for nonproportional hazards. The challenge in moving away from proportional hazards is not so much computational, as interpretational, as there is no single number summarizing the difference between the groups. The restricted mean survival time is one alternative, but this requires agreement on the “event horizon.”

In conclusion, we believe reference‐based sensitivity analysis via multiple imputation is a flexible, accessible, and practical approach, as witnessed by its increasing use. We hope that, by showing how these ideas can be extended to survival data, practitioners will have confidence to use it in their own studies.
